# From raw material to functional food: Effect of dry-heat treatment on whole quinoa flour structure and dough rheology, with in vivo hypoglycemic validation at the optimal temperature

**DOI:** 10.1016/j.fochx.2026.104360

**Published:** 2026-08-30

**Authors:** Ya-li Zhou, Xiao-long Li, Xin-yong You, Xing-yun Wang

**Affiliations:** College of Biological and Food Engineering, Anyang Institute of Technology, Anyang 455000, Henan, China

**Keywords:** Quinoa flour, Dry-heat treatment, Starch digestibility, Hypoglycemic effect, Dough rheology

## Abstract

This study investigated the effects of dry-heat treatment (DHT) temperature on whole quinoa flour structure, dough rheology, bread texture, starch digestibility, and hypoglycemic potential in STZ-induced diabetic mice. DHT induced surface aggregate detachment and imperfections on flour particles, while preserving the A-type starch crystallinity. Compared to untreated controls, DHT (110–150 °C) increased dough water absorption and softening degree, but decreased extensibility and resistance. Bread made with 110 °C-treated flour showed a slight decrease in hardness and a significant improvement in springiness; higher temperatures progressively deteriorated the texture. DHT notably lowered rapidly digestible starch and elevated slowly digestible and resistant starch fractions. Based on these in vitro results, 110 °C was identified as the optimal temperature, and only the 110 °C-treated flour was used for in vivo evaluation. In STZ-induced diabetic mice, six-week quinoa bread supplementation suggested beneficial effects on glycated hemoglobin, blood glucose, and insulin levels. These findings provide a theoretical basis for designing quinoa-based functional foods.

## 1 Introduction

Quinoa (*Chenopodium quinoa* Willd.), a pseudocereal indigenous to South America, has gained global agricultural importance due to its remarkable resilience to abiotic stresses such as salinity, drought, and acidic soils. Its cultivation has expanded worldwide, including regions of the United States, Canada, India, Italy, and China (Xing et al., 2023). In recent years, quinoa has attracted significant research interest not only for its agronomic adaptability but also for its exceptional nutritional profile. It contains a high protein content (14–18%) with a balanced amino acid composition that aligns with the FAO recommendations, being particularly rich in the essential amino acids histidine, lysine, threonine, and methionine, which are often limiting in common cereals (Ren et al., 2023). Furthermore, quinoa is a valuable source of dietary fiber, vitamins, minerals, and bioactive phytochemicals including polyphenols and phytosterols (Ren et al., 2023; Yang et al., 2024). These bioactive compounds contribute to its potent antioxidant capacity and are associated with potential cardiometabolic benefits, including anti-diabetic properties (Jan et al., 2023; Kwa´ sniewska et al., 2023). Notably, quinoa is gluten-free, alkaline, and has a low glycemic index (GI), positioning it as an excellent raw material for developing functional foods (Rocchetti et al., 2019). Despite its nutritional advantages, the widespread consumption of wheat bread—a dietary staple with a high glycemic response—poses a health concern related to postprandial blood glucose management (Ballester-Sánchez et al., 2019). Fortifying bread with quinoa flour has been shown to enhance its polyphenol content and antioxidant activity (Brend et al., 2012). However, the specific impact of quinoa incorporation on the digestibility of bread starch and its consequent effect on postprandial glycemia remains inadequately explored.

Starch, the predominant component of bread, is a major determinant of its glycemic response (Wang et al., 2024). Excessive intake of rapidly digestible starch can lead to undesirable postprandial glucose spikes and long-term metabolic risks. Modifying starch digestibility through physical or chemical means offers a viable strategy to improve the nutritional profile of starchy foods. Various physical and chemical methods, such as octenyl succinylation (Kurichh et al., 2025), annealing and heat-moisture treatment (Sang et al., 2025), ultrasonication (Awais et al., 2025), and acid modification (Mojo-Quisani et al., 2024), have been employed to retard starch hydrolysis. Among these, dry-heat treatment (DHT) defined as the heating of cereals or starch with a moisture content below 10% at temperatures between 110 and 150 °C for a controlled duration—stands out for its operational simplicity, cost-effectiveness, and absence of chemical residues. DHT preserves granular integrity while markedly altering the physicochemical and functional properties of starch (He et al., 2025). Previous studies report divergent effects of DHT on starch digestibility, which appear to be source dependent. For instance, DHT increased resistant starch content in rice (Oh et al., 2018) and barnyard millet (Praba et al., 2019), but elevated rapidly digestible starch in chestnut (Fang et al., 2025) and quinoa starch (Zhou et al., 2020). This variability underscores that the outcome of DHT is highly influenced by the botanical source and specific treatment parameters. Although DHT has been applied to modify flour functionality in breadmaking, its targeted use for modulating the digestibility and hypoglycemic potential of quinoa-enriched bread has not been systematically investigated.

While the effects of DHT have been studied in various cereal and starch systems, a comprehensive understanding of how DHT temperature modulates the multiscale structure of whole quinoa flour and, consequently, the technological performance, in vitro digestibility, and in vivo glycemic response of quinoa-wheat mixed bread is lacking. The present study treated whole quinoa flour at 110 °C, 130 °C, and 150 °C and characterized the resulting structural modifications using scanning electron microscopy (SEM), X-ray diffraction (XRD), and Fourier-transform infrared (FTIR) spectroscopy. The effects on mixed dough rheology (assessed by farinograph and extensograph) and bread texture were evaluated. In vitro starch digestion was analyzed to quantify RDS, slowly digestible starch (SDS), and RS fractions. Finally, the hypoglycemic potential of the breads was examined in a diabetic mouse model.

To extend the scope of quinoa-based functional food research, this study adopted a two-stage “in vitro screening → in vivo validation” strategy. Such a hierarchical design is widely recommended in functional food research to minimize animal usage, reduce experimental costs, and improve research efficiency, while ensuring that only the most promising candidate is carried forward to animal trials (Fujiwara et al., 2017). In the first stage, three DHT temperatures (110, 130, and 150 °C) were compared for their effects on flour structure, dough rheology, and in vitro starch digestibility to identify the optimal treatment. In the second stage, only the optimal temperature was used to prepare bread for in vivo hypoglycemic assessment in a diabetic mouse model. This approach not only adheres to the 3Rs principle (Replacement, Reduction and Refinement) in animal experimentation but also provides a clear, evidence-based rationale for the selection of the test condition in the animal study, thereby enhancing the transparency and scientific rigor of the experimental design. By integrating structural, technological, and biological analyses, this work aims to establish a clear structure–function relationship and provide a scientific basis for developing quinoa-based functional breads with tailored digestibility and enhanced metabolic benefits.

## 2 Materials and methods

### 2.1 Material

Quinoa seeds were obtained from Germu Namulan Trading Co., Ltd. (Qinghai, China). The proximate composition (dry basis) was as follows: starch 67.32%, protein 15.48%, fat 6.02%, ash 2.32%, and moisture 9.64%. High-gluten wheat flour (bread flour) was purchased from Hebei Qiusu Food Sales Co., Ltd. (Hebei, China), with a composition of starch 75.21%, protein 10.92%, fat 1.62%, ash 0.62%, and moisture 11.34%. Instant dry yeast was provided by Lesaffre (Mingguang) Co., Ltd. (Anhui, China). Petroleum ether and sodium hydroxide were acquired from Tianjin Huihang Chemical Technology Co., Ltd. (Tianjin, China). Streptozotocin was sourced from Shanghai Yuanye Bio-Technology Co., Ltd. (Shanghai, China). Male C57BL/6 mice were supplied by Henan Kebes Biotechnology Co., Ltd. (Laboratory Animal Production License No. SCXK (Yu) 2020–0005). Commercial ELISA kits for insulin (CEA448Mu) and mouse glycated hemoglobin A1c (GHbA1c; SEA928Mu) were obtained from Wuhan Servicebio Biotechnology Co., Ltd. (Hubei, China). Standard rodent diet was provided by Jiangsu Xiehe Medical and Biological Engineering Co., Ltd. (Production License No. Su Si Zheng (2019) 01008).

### 2.2 Preparation and dry-heat treatment of whole quinoa flour

Quinoa seeds with plump and intact grains were selected. Impurities and moldy seeds were removed, and the seeds were washed with distilled water. They were then dried in an oven at 40 °C until the moisture content fell below 10%. The dried seeds were ground using a pulverizer and passed through a 100-mesh standard sieve. The resulting whole quinoa flour was evenly divided into four portions. Three portions were subjected to dry-heat treatment in an oven at 110 °C, 130 °C, and 150 °C, respectively, for 1 h. After treatment, the samples were cooled to room temperature and stored in sealed containers. The remaining portion, without any dry-heat treatment, served as the control.

### 2.3 Process for quinoa bread production

Toast bread was prepared using the sponge and dough method. The base formula for the control bread (0% quinoa) was as follows: Sponge dough: bread flour 196 g, water 120 g, salt 3 g, active dry yeast 2 g, milk powder 10 g. Main dough: bread flour 104 g, water 50 g, salt 1 g, active dry yeast 1 g, white sugar 15 g, olive oil 15 g. For quinoa-enriched bread, 15% of the total wheat flour (i.e., 45 g per 300 g of the flour blend) was replaced with whole quinoa flour (either untreated or DHT-treated at 110, 130, or 150 °C), while all other ingredients remained identical to the control formulation. Bread samples prepared with untreated quinoa flour and quinoa flour treated at 110 °C, 130 °C, and 150 °C were designated as Control, 110 °C, 130 °C, and 150 °C, respectively.

The key processing parameters were as follows: The dough was mixed in a mixer at low speed for 2 min, followed by high-speed mixing until full gluten development (approximately 8–10 min). Fermentation (including resting and proofing) was conducted at 30 °C and 75% relative humidity for a total time of 90 min. Baking was carried out in a preheated oven at 180 °C (top heat) and 200 °C (bottom heat) for 25 min. The baked bread was cooled to room temperature prior to further analysis.

### 2.4 Structural characterization of whole quinoa flour

#### 2.4.1 Scanning electron microscopy (SEM)

The morphological structure of quinoa flour samples was analyzed using a scanning electron microscope. Images were acquired at an accelerating voltage of 5.0 kV and a magnification of 3000 × .

#### 2.4.2 X-ray diffraction (XRD)

X-ray diffraction patterns were obtained using a Smartlab SE X-ray diffractometer (Rigaku, Japan) operated in step-scan mode. The measurement conditions were as follows: Cu-Kα radiation, tube voltage of 40 kV, tube current of 100 mA, scanning range (2θ) from 4° to 60°, step size of 0.02°, and scanning speed of 6°/min. The raw data were processed using File Exchange V7 software. The relative crystallinity of the whole quinoa flour was calculated as the ratio of the crystalline peak area to the total area after background subtraction using Jade 6.0 software (Wang et al., 2020).

#### 2.4.3 Fourier transform infrared (FT-IR) spectroscopy

Fourier transform infrared spectroscopy was employed to analyze both native and dry-heat-treated quinoa flour samples. Samples were prepared by thoroughly mixing the flour with KBr at a ratio of 1:100 (mg/mg) and pressing into thin pellets. Spectra were recorded in the wavenumber range of 4000–400 cm^−1^ with a spectral resolution of 4 cm^−1^, accumulating 64 scans per sample.

### 2.5 Determination of farinograph properties of quinoa-wheat mixed dough

The farinograph properties of the quinoa-wheat mixed dough were determined following the method described in the Chinese National Standard GB/T 14614–2019 (GB/T14614–2019, 2019; Guo et al., 2023). A 300 g sample of mixed flour, consisting of 85% wheat flour and 15% whole quinoa flour treated at different dry-heat temperatures, was used for each test (the same substitution ratio as used in the bread formulation). The dough containing untreated quinoa flour served as the control (designated as Control), while doughs containing quinoa flour treated at 110 °C, 130 °C, and 150 °C were designated as 110 °C, 130 °C, and 150 °C, respectively. The flour sample was placed into the mixing bowl, and the instrument (Farinograph) was started to begin the measurement. The water absorption, dough development time, stability time, and degree of softening of the mixed dough were recorded. Each experiment was performed in triplicate.

### 2.6 Determination of extensograph properties of quinoa-wheat mixed dough

The extensograph properties were determined according to the method specified in the Chinese National Standard GB/T 14615–2019 (GB/T14615–2019, 2019; Guo et al., 2023). The test was performed using a Spaghetti/Noodle Tensile Rig (Code A/SPR) probe. The instrument parameters were set as follows: pre-test speed 2 mm/s, test speed 2 mm/s, post-test speed 10 mm/s, test distance 100 mm, and probe type Auto-0.5 g. A 300 g sample of composite flour, consisting of 85% wheat flour and 15% whole quinoa flour subjected to different dry-heat treatments, was used for each test. The dough containing untreated quinoa flour served as the control. After proofing, the dough was placed on the extensograph platform. The dough was stretched until rupture to obtain the extensogram. Each measurement was performed in triplicate.

### 2.7 Analysis of texture profile of quinoa bread

The texture profile analysis (TPA) was performed following the method of Yang and Zhao (2020) with slight modifications. A P/36R cylindrical probe was used under the following settings: test speed 30 mm/s, trigger load 0.1 N, and compression ratio 80%. Hardness and springiness of quinoa bread were determined, with three replicates per sample.

### 2.8 In vitro starch digestibility

In vitro starch digestion was conducted according to the Englyst et al. (1992) with modifications. Briefly, 100 mg of quinoa bread sample (designated as G0, G20, and G120) was weighed into a 50 mL centrifuge tube. Then, 10 mL of 0.5 mol/L sodium acetate-acetic acid buffer (pH 5.2) was added, and the mixture was equilibrated in a shaking water bath at 37 °C for 10 min. Subsequently, 1 mL of amyloglucosidase (2500 U/mL) and 4 mL of porcine pancreatic α-amylase were added. For the G₀ sample, the reaction was terminated immediately after adding the enzyme solution by placing the tube in a boiling water bath and heating for 5 min. The reaction mixture was shaken at 200 r/min, incubated for 20 min or 120 min, and the reaction was immediately terminated by boiling for 5 min. After cooling, the mixture was centrifuged at 3500 r/min for 10 min. The supernatant was collected, and the pellet was re-extracted under the same conditions. The combined supernatants were transferred to a 100 mL volumetric flask and made up to volume. Finally, 1 mL of the diluted solution was taken for reducing sugar determination using the DNS method.

The percentage of starch hydrolysis was calculated as follows:

$$\mathrm{RDS}\left(\%\right)=\frac{\left(G_{20}-G_{0}\right)\times 0.9}{\mathrm{TS}}\times 100\%$$

$$\mathrm{SDS}\left(\%\right)=\frac{\left(G_{120}-G_{20}\right)\times 0.9}{\mathrm{TS}}\times 100\%$$

$$\mathrm{RS}\left(\%\right)=1-\frac{\left(\mathrm{RDS}+\mathrm{SDS}\right)}{100}\times 100\%$$

where G₀, G₂₀, and G₁₂₀ represent the reducing sugar content in the hydrolysate after 0, 20, and 120 min of digestion, respectively, and TS is the total starch content of the sample (mg); and 0.9 is the conversion factor from glucose to starch (162/180). RDS, SDS, and RS represent rapidly digestible starch, slowly digestible starch, and resistant starch, respectively.

### 2.9 Determination of physicochemical quality indicators of quinoa bread

The physicochemical analysis of quinoa bread was conducted in accordance with the Chinese National Food Safety Standards, 5009.3–2016 (moisture), 5009.4–2016 (ash), 5009.5–2025 (protein), and 5009.88–2023 (5009.88–2023), respectively.

### 2.10 Preparation of phenolic extract from quinoa bread

Approximately 1.00 g of dried bread sample was immersed in 100 mL of 75% ethanol solution. The mixture was kept in a constant-temperature water bath at 40 °C for 2 h, then centrifuged at 5000 r/min for 5 min at 4 °C. The supernatant was collected, cooled, concentrated, and finally made up to 20 mL with ethanol.

#### 2.10.1 Determination of total phenolic content

The total phenolic content was measured according to the method described by Amiri et al. (2023) with slight modifications. Briefly, 1 mL of the sample extract was mixed with 1 mL of Folin-Ciocalteu reagent and 3 mL of distilled water in a 50 mL centrifuge tube. After standing at room temperature for 5 min, 5 mL of 7.5% Na₂CO₃ solution was added and mixed thoroughly. The mixture was kept in the dark at room temperature for 90 min, and the absorbance was measured at 765 nm using a spectrophotometer. A standard curve was prepared using gallic acid solutions at different concentrations. The absorbance at 765 nm was recorded, and the standard curve was plotted with gallic acid content as the abscissa and absorbance (A) as the ordinate. The regression equation obtained was y = 0.0022× + 0.001 (R^2^ = 0.9997). Based on this standard curve, the total phenolic content in the samples was calculated and expressed as mg gallic acid equivalent per gram dry weight (mg GAE/g DW). All assays were performed in triplicate.

#### 2.10.2 DPPH radical scavenging activity assay

The DPPH radical scavenging activity was determined according to the method described by Yesil and Levent (2022) with slight modifications. Briefly, 2.0 mL of the sample extract was pipetted into a test tube and mixed with 2.0 mL of DPPH-ethanol solution (0.1 mmol/L). The mixture was kept in the dark for 15 min, and the absorbance was measured at 517 nm (recorded as A₁). For the blank control, ethanol was used instead of the DPPH-ethanol solution, and the absorbance was measured under the same conditions (recorded as A₂). A control was prepared by replacing the sample extract with ethanol, and its absorbance was measured (recorded as A₀). The DPPH radical scavenging rate was calculated using the following formula:

$$\text{DPPH radical scavenging rate}\;\left(\%\right)=\left[1–\left(A₁–A₂\right)/A₀\right]\times 100$$

### 2.11 Animal experiment

#### 2.11.1 Establishment and maintenance of mouse model

Male C57BL/6 mice weighing 20 ± 2 g were used in this study. The mice were housed under controlled conditions (temperature: 23 ± 2 °C; relative humidity: 50–60%) with a 12 h light/dark cycle and had free access to food and water. After a one-week acclimatization period, eight mice were randomly selected as the normal control group. Diabetes was induced by a single intraperitoneal injection of streptozotocin (STZ) at a dose of 125 mg/kg body weight per day. Prior to injection, the mice were fasted for 12 h (water was allowed). The normal control group received an equal volume of 0.1 mol/L citrate buffer (pH 4.4). One week after STZ induction, mice in the treatment groups exhibited typical diabetic symptoms, including polydipsia, polyuria, and reduced body weight gain. Fasting blood glucose (FBG) levels were measured via tail vein sampling after a 12-h fast. Mice with FBG levels between 10 and 25 mmol/L were considered successfully modeled. Mice with FBG levels below 10 mmol/L or above 25 mmol/L, as well as those showing severe weight loss (>20% of initial body weight), were excluded from the study to ensure model consistency.

The successfully modeled diabetic mice were then randomly divided into four groups (*n* = 8 per group): the diabetic model group, the quinoa flour group, the quinoa bread group (containing 15% quinoa flour), and the normal control group. There was no significant difference in baseline FBG levels among the three diabetic groups (*P* > 0.05), confirming the homogeneity of the groups before intervention. The quinoa flour group (QF) was fed dough blocks prepared by mixing 110 °C dry-heat-treated quinoa flour with distilled water at a ratio of 2:1 (flour:water, *w*/w) to form a cohesive dough. The dough was freshly prepared every three days and stored at 4 °C until use. Each mouse was provided with approximately 3 g of dough per day (equivalent to approximately 2 g of quinoa flour). The quinoa bread group (QB) was fed bread prepared with 15% 110 °C dry-heat-treated quinoa flour (as described in Section 2.3). The bread was sliced into small pieces (approximately 1 cm^3^) and dried at 40 °C for 12 h to facilitate feeding and storage. Each mouse was provided with approximately 3.5 g of dried bread per day. The diabetic model group and the normal control group were fed a standard rodent diet. At the end of the 6-week intervention period, all mice were euthanized by cervical dislocation under isoflurane anesthesia to minimize suffering. This method was performed in accordance with the guidelines approved by the Institutional Animal Care and Use Committee of the Institute of Radiation Medicine, Chinese Academy of Medical Science (Approval No. IRM-DWLL-2022250). All groups had free access to water. The standard rodent diet (Normal and Model groups) was provided as pelleted feed, while the Quinoa flour and Quinoa bread diets were provided in separate feeding containers to prevent cross-contamination and allow for accurate measurement of food consumption.

#### 2.11.2 Determination of basic physicochemical and biochemical parameters

Throughout the feeding period, mouse body weight, food intake, and fasting blood glucose (FBG) levels were measured every three days. Prior to each measurement, mice were fasted for 12 h with free access to water. For FBG measurement, a blood sample was collected from the tail vein using a blood glucose test strip to absorb one drop of naturally flowing blood, and the blood glucose level was immediately determined using a glucometer. Fasting insulin (FINS) levels and glycated hemoglobin (GHb) content were measured using the respective commercial assay kits strictly according to the manufacturers' instructions.

### 2.12 Data analysis

The experimental data were initially processed using Microsoft Office Excel 2010. Statistical analysis was performed with SPSS 17.0 software. All data were subjected to one-way analysis of variance (ANOVA) for significance testing. If significant differences were detected (*P* < 0.05), Duncan's multiple range test was further applied for post hoc comparisons between groups. For in vitro experiments (including farinograph, extensograph, texture analysis, in vitro starch digestibility, and physicochemical determinations), data are presented as mean ± standard deviation (SD) with *n* = 3 technical replicates (three independent experimental runs). For the animal experiment, data are presented as mean ± standard error of the mean (SEM) with *n* = 8 biological replicates (eight mice per group). All graphs were plotted using Origin 2024 software.

## 3 Results and analysis

### 3.1 Effects of dry-heat treatment temperature on the structure of quinoa flour

#### 3.1.1 Analysis of granular morphological structure

**Fig. 1:**
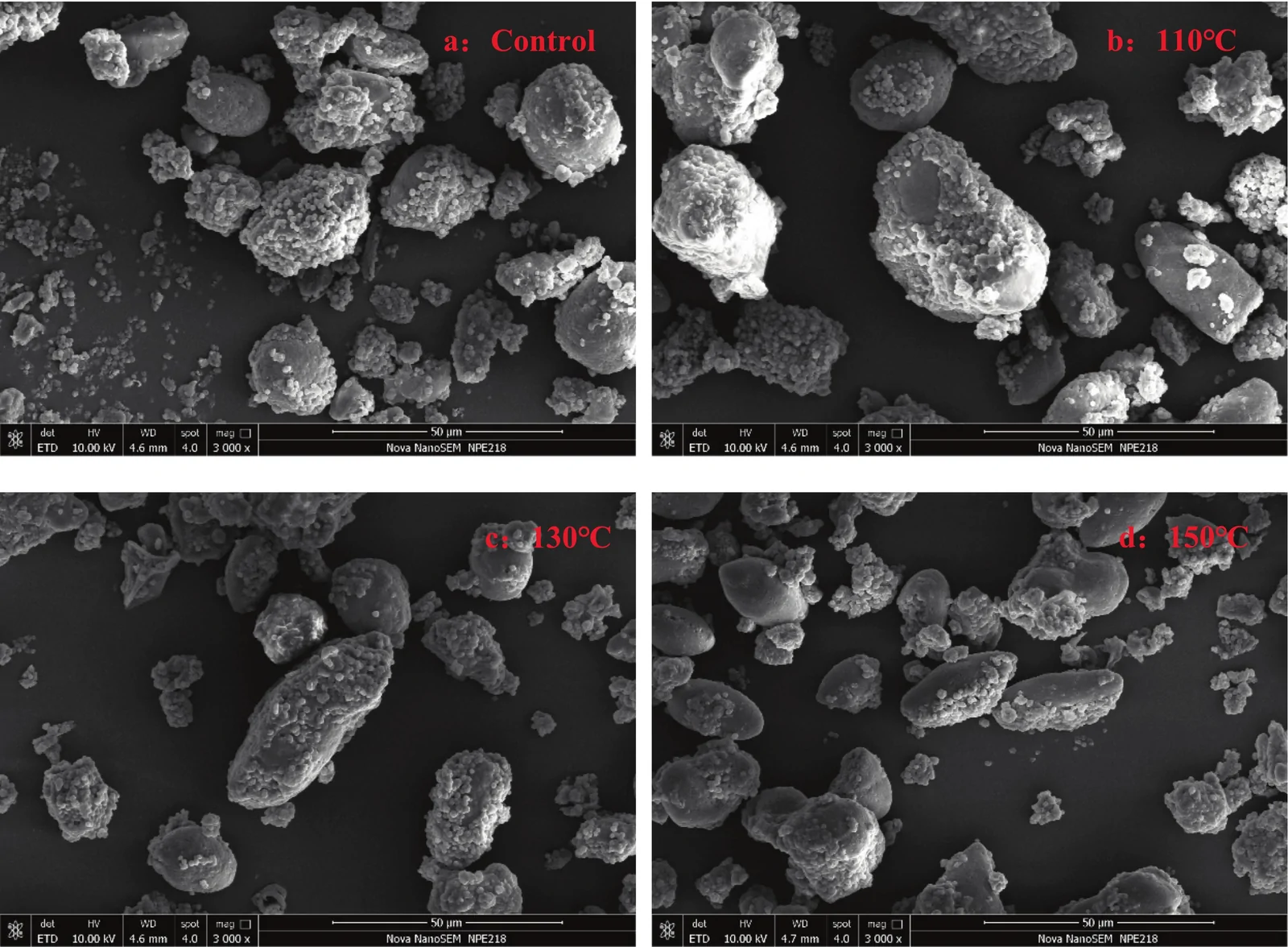
Scanning electron microscopy (SEM) images of whole quinoa flour samples subjected to different dry-heat treatment (DHT) temperatures. (a) Control (untreated); (b) 110 °C (treated at 110 °C for 1 h); (c) 130 °C (treated at 130 °C for 1 h); (d) 150 °C (treated at 150 °C for 1 h). The flour particles exhibited spherical to ellipsoidal morphologies with surface protein aggregates. DHT induced progressive detachment of these surface aggregates and increased surface roughness with increasing temperature. Magnification: 3000×; accelerating voltage: 5.0 kV; scale bar = 10 μm.

As shown in Fig. 1, whole quinoa flour particles exhibited spherical to ellipsoidal morphologies. The particle surfaces were coated with abundant protein bodies, forming a distinct aggregated surface layer, a microstructure fundamentally similar to that observed in wheat flour particles. Compared to untreated flour, dry-heat treatment (DHT) induced noticeable surface defects and promoted the detachment of these proteinaceous aggregates. The extent of detachment increased progressively with treatment temperature. These morphological alterations can be attributed to the thermomechanical disruption of intermolecular interactions within the flour matrix. Specifically, DHT is known to weaken hydrogen bonding between starch chains, leading to partial disorganization and fracture of starch granules. Concurrently, elevated temperatures promote the denaturation of surface-associated proteins, reducing their adhesive capacity and compromising the overall structural integrity of the particles. This combined effect, starch granule perturbation and protein matrix disintegration, likely underpins the observed increase in surface roughness and aggregate loss (González et al., 2020; Wang et al., 2025). Such structural modifications at the particle level are expected to directly influence subsequent dough rheology, and ultimately, the functional properties of the derived bread.

#### 3.1.2 X-ray diffraction analysis

**Fig. 2:**
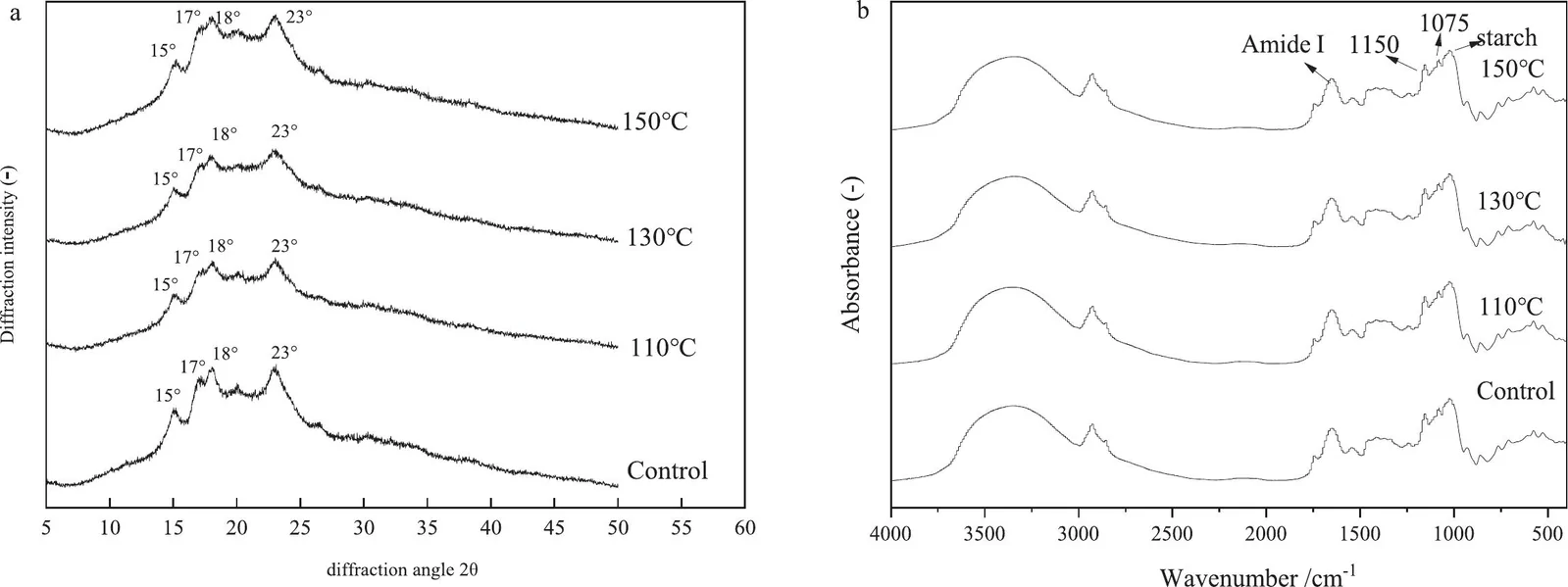
(a) X-ray diffraction (XRD) patterns and (b) Fourier-transform infrared (FT-IR) spectra of whole quinoa flour samples subjected to different dry-heat treatment (DHT) temperatures. Characteristic diffraction peaks at 2θ ≈ 15°, 17°, 18°, and 23° indicate a typical A-type crystalline structure and the major FT-IR absorption regions (amide I band at 1700–1600 cm^−1^ and carbohydrate backbone at 1060–960 cm^−1^). Control, untreated quinoa flour; 110, 130, and 150 denote quinoa flour treated at 110 °C, 130 °C, and 150 °C for 1 h, respectively.

The XRD patterns of quinoa flour subjected to various dry-heat treatments are presented in Fig. 2a. Characteristic diffraction peaks were observed at approximately 15°, 17°, 18°, and 23°, corresponding to a typical A-type crystalline structure, which is common in cereal starches (Zhou et al., 2020). No notable shift, disappearance, or appearance of new peaks was detected with increasing treatment temperature, indicating that the dry-heat treatment did not alter the fundamental crystalline polymorph of the starch. This finding is consistent with previous reports (Zhou et al., 2020). However, as summarized in Table S1, the relative crystallinity of the flour increased significantly after dry-heat treatment. Compared with the control (untreated), the crystallinity rose by 7.24%, 10.68%, and 12.06% for samples treated at 110 °C, 130 °C, and 150 °C, respectively. This increase in crystallinity can be explained by the reorganization of starch molecules within the granules during thermal treatment. The applied heat likely facilitates the mobility of amylose and amylopectin chains, allowing them to re-align into more ordered, crystalline domains. Simultaneously, the partial detachment of surface proteins (as observed in SEM) may reduce steric hindrance at the granule surface, further promoting the rearrangement and perfection of the crystalline regions. Such structural consolidation enhances the molecular order and contributes to the increased resistance to enzymatic digestion, as reflected in the subsequent in vitro digestibility results (González et al., 2020; Wang et al., 2025; Zhou et al., 2020).

#### 3.1.3 Fourier transform infrared (FT-IR) spectroscopy analysis

FT-IR spectroscopy provides insight into the short-range molecular order and the chemical environment of functional groups in starch granules, which is influenced by the helical conformation of amylose and amylopectin. The spectra of native and dry-heat-treated quinoa flour samples across the range of 4000–400 cm^−1^ are displayed in Fig. 2b. Two prominent absorption bands were identified: a broad band around 1700–1600 cm^−1^, characteristic of the amide I vibration primarily arising from protein components, and a strong band between 1060–960 cm^−1^, attributed to C—O and C—C stretching vibrations of the carbohydrate backbone in starch (Bai et al., 2022; González et al., 2020). Additional weaker signals near 1150 cm^−1^ and 1075 cm^−1^ are associated with hemicellulose, cellulose, and pectin present in the whole flour matrix (Ying et al., 2017). Critically, no new absorption peaks emerged, and no existing characteristic peaks disappeared following dry-heat treatment, indicating that the treatment did not induce the formation or cleavage of covalent bonds or significantly alter the primary chemical structure of the major flour constituents. This structural stability can be attributed to the nature of dry-heat treatment, which operates under low moisture conditions (<10%) and moderate temperatures (110–150 °C). Under these conditions, the predominant effects are physical and involve the disruption of non-covalent interactions (e.g., hydrogen bonds and hydrophobic forces), as evidenced in SEM and XRD analyses, rather than chemical reactions such as decomposition or Maillard browning. The preservation of key chemical groups confirms that DHT is a mild physical modification method that maintains the native biochemical integrity of quinoa flour while altering its higher-order structure. This retained chemical profile suggests that the functional and nutritional properties associated with these native components, such as protein quality and inherent antioxidant capacity, are likely preserved, providing a stable material base for subsequent dough formation and baking.

### 3.2 Effect of dry-heat treatment temperature on the farinograph properties of mixed dough

As shown in Table 1, the water absorption of the mixed dough increased significantly with increasing dry-heat treatment temperature. Compared to the control dough containing untreated quinoa flour, the incorporation of flour treated at 110 °C raised water absorption by 1.79%, with a maximum increase of 4.83% observed at 150 °C. This enhancement suggests that dry-heat treatment improves the water-holding capacity of the composite system. The increase may be attributed not only to the thermal modification of starch and protein structures but also to the higher cellulose content introduced by quinoa flour, which promotes water binding (Hamzehpour & Dastgerdi, 2023). Elevated water absorption is often associated with improved bread yield. In contrast, dough development time exhibited a decreasing trend as treatment temperature increased. At 110 °C, development time was reduced by 8.89%. This reduction likely results from the partial disruption of the gluten network in the wheat-quinoa blend during heating, which diminishes both gluten quantity and quality. Similarly, stability time decreased markedly by 16.44% at 110 °C, reaching its lowest value at 150 °C. These changes indicate that dry-heat treatment interferes with the formation of robust protein-peptide interactions and an ordered, continuous network, thereby compromising dough stability. The degree of softening rose substantially with increasing treatment temperature. Relative to the control, softening increased by 43.75% at 110 °C and reached a maximum increase of 125% at 150 °C. Softening is closely linked to gluten content and strength. Since quinoa flour is gluten-free, its incorporation dilutes the overall gluten protein in the composite dough. Moreover, dry-heat treatment further weakens the existing gluten network and induces starch-granule fracture, collectively reducing dough strength and tenacity.

**Table 1:** Effect of different dry-heat treatment (DHT) temperature on farinograph properties of mixed dough.

Index	dry-heat treatment temperature/°C			
	Control	110 °C	130 °C	150 °C
Water absorption/%	61.50 ± 0.46^d^	62.60 ± 0.23^bc^	63.50 ± 0.17^ab^	64.47 ± 0.18^a^
Development time/min	4.50 ± 0.07^a^	4.10 ± 0.04^ab^	3.20 ± 0.05^c^	2.40 ± 0.08^d^
Stability time/min	14.60 ± 0.33^a^	12.20 ± 0.39^b^	9.50 ± 0.25^c^	7.60 ± 0.19^d^
Softening/FU	48.00 ± 1.33^d^	69.00 ± 1.36^c^	98.00 ± 2.58^b^	108.00 ± 3.88^a^

Control, untreated quinoa flour; 110 °C, 130 °C, and 150 °C denote quinoa flour dry-heat treated at 110 °C, 130 °C, and 150 °C for 1 h, respectively. Values are expressed as mean ± standard deviation (*n* = 3). Different lowercase letters within the same row indicate significant differences among treatment groups (*P* < 0.05, one-way ANOVA followed by Duncan's multiple range test).

### 3.3 Effect of dry-heat treatment temperature on the extensograph properties of mixed dough

The extensibility of the mixed dough showed a numerical decrease with increasing dry-heat treatment temperature, although the differences were not statistically significant. At 90 and 135 min, the extensibility decreased significantly (Table 2). Compared to the control, the use of quinoa flour treated at 110 °C resulted in reductions in extensibility of 10.91%, 10.00%, and 11.15% after 45, 90, and 135 min of proofing, respectively. This decrease can be primarily attributed to the dilution of gluten in the wheat-quinoa blend. As a gluten-free ingredient, quinoa flour lowers the overall concentration of gluten-forming proteins (gliadin and glutenin), thereby diminishing the dough's capacity for stretching. Both the maximum resistance and the resistance to extension also exhibited a marked decline with increasing treatment temperature at each proofing interval. For any given treatment temperature, these resistance values increased with longer proofing times (45 min < 90 min < 135 min), yet the absolute values remained lower than those of the control. This pattern confirms that elevated dry-heat temperatures generally weaken the structural strength of the dough.

**Table 2:** Effect of different dry-heat treatment (DHT) temperature on extensograph properties of mixed dough.

Index		dry-heat treatment temperature/°C			
		Control	110 °C	130 °C	150 °C
Extensibility/mm	45 min	128.33 ± 4.63^Aa^	114.33 ± 6.36^Ba^	108.0 ± 3.21^Ca^	100.33 ± 2.03^Ca^
	90 min	120.00 ± 3.06^Aa^	108.00 ± 2.52^Bb^	98.33 ± 1.45^Ca^	63.67 ± 1.20^Dc^
	135 min	98.67 ± 1.76^Ab^	87.67 ± 2.33^Bc^	80.33 ± 1.48^Cb^	73.67 ± 1.86^Db^
Maximum resistance /EU	45 min	650.33 ± 7.14^Ac^	629.33 ± 1.41^Bc^	600.67 ± 9.1^Cc^	474.33 ± 7.70^Db^
	90 min	834.0 ± 1.82^Ab^	814.67 ± 1.62^Bb^	797.00 ± 9.66^Cb^	634.33 ± 6.12^Da^
	135 min	898 ± 5.13^Aa^	854.67 ± 5.96^Ba^	806.33 ± 3.54^Ca^	644.670 ± 9.21^Da^
Resistance/EU	45 min	486.33 ± 3.48^Ac^	465.33 ± 4.33^Bc^	441.67 ± 6.64^Cc^	397.67 ± 2.17^Dc^
	90 min	673.33 ± 6.36^Aa^	604.0 ± 7.23^Bb^	574.67 ± 4.06^Cb^	487.67 ± 4.10^Db^
	135 min	567.00 ± 3.79^Cb^	625.33 ± 4.91^Ba^	696.0 ± 9.07^Aa^	502.67 ± 6.64^Da^
Resistance/Extensibility ratio	45 min	3.78 ± 0.02^Cb^	4.06 ± 0.04^Ac^	4.04 ± 0.04^Ac^	3.92 ± 0.05^Bc^
	90 min	5.71 ± 0.02^Ba^	5.86 ± 0.03^Ab^	5.67 ± 0.03^Cb^	5.26 ± 0.03^Db^
	135 min	5.79 ± 0.01^Da^	8.66 ± 0.03^Aa^	7.18 ± 0.02^Ba^	6.71 ± 0.05^Ca^

Control, untreated quinoa flour; 110 °C, 130 °C, and 150°C denote quinoa flour dry-heat treated at 110 °C, 130 °C, and 150 °C for 1 h, respectively. Values are expressed as mean ± SD (*n* = 3). Different uppercase letters (A, B, C) in the same row indicate significant differences among different treatment groups at the same time point (*P* < 0.05). Different lowercase letters (a, b, c) in the same column indicate significant differences among different time points within the same treatment group (*P* < 0.05). Control, untreated quinoa flour; 110 °C, 130 °C, and 150 °C denote quinoa flour dry-heat treated at 110 °C, 130 °C, and 150 °C for 1 h, respectively.

The resistance-to-extensibility ratio, an indicator of dough tenacity, initially rose and then fell as treatment temperature increased. The highest ratios were observed for dough containing 110 °C-treated flour, reaching 4.06, 5.86, and 8.66 after 45, 90, and 135 min of proofing, respectively. The initial increase may reflect a moderate thermal modification that temporarily stabilizes protein interactions. However, at higher temperatures, the disruption of cross-linking bonds that maintain protein tertiary structure becomes more pronounced, leading to a loss of stability and a consequent decline in both resistance and the ratio. Since a higher resistance-to-extensibility ratio typically denotes stronger gluten quality, the composite dough with quinoa flour treated at 110 °C demonstrated the most desirable balance of strength and extensibility in this study.

### 3.4 Effect of dry-heat treatment temperature on the textural properties of bread

**Fig. 3:**
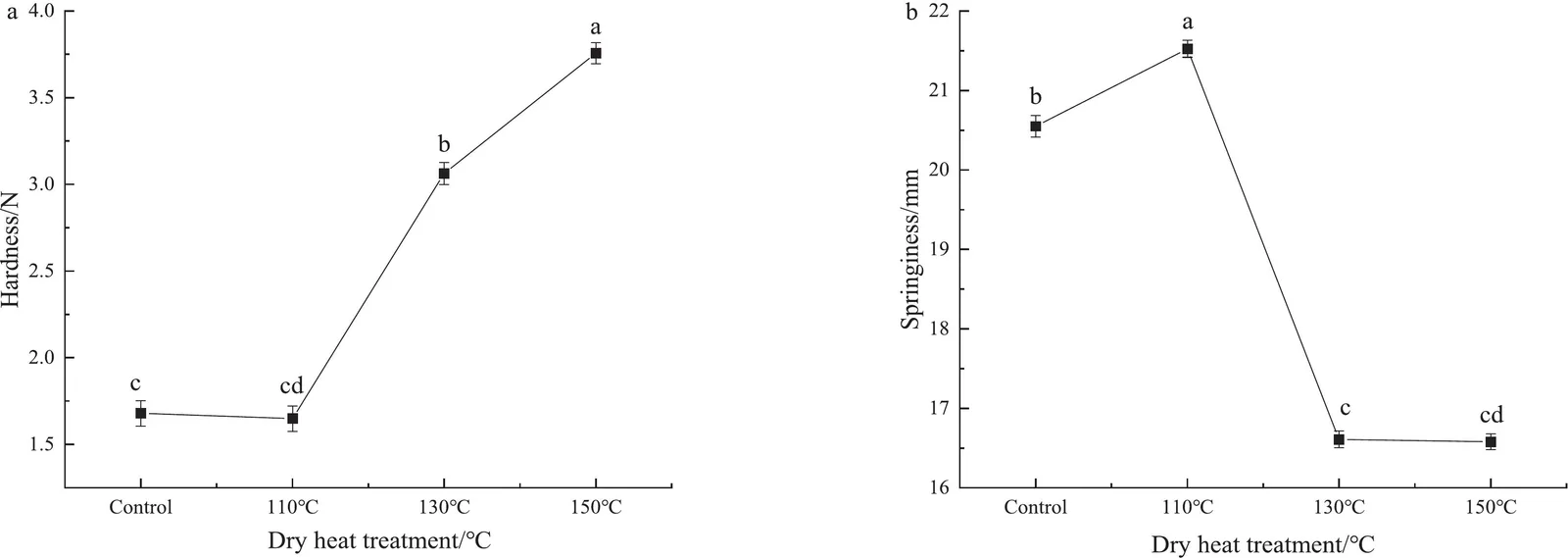
Effects of different dry-heat treatment (DHT) temperature on (a) hardness and (b) springiness of quinoa bread. Control, untreated quinoa flour; 110 °C, 130 °C, and 150 °C denote quinoa flour treated at 110 °C, 130 °C, and 150 °C for 1 h, respectively. Values are expressed as mean ± SD (*n* = 3). Different lowercase letters above the bars indicate significant differences among treatment groups (*P* < 0.05, one-way ANOVA followed by Duncan's multiple range test).

As illustrated in Fig. 3, the hardness of quinoa-enriched bread decreased slightly at 110 °C and then progressively increased at higher dry-heat treatment temperature. The lowest hardness value was observed in bread prepared with flour treated at 110 °C, which was 1.82% lower than the control, however, the difference was not statistically significant (*P* > 0.5). In contrast, bread containing flour treated at 150 °C exhibited the highest hardness. Springiness showed a different trend, reaching a maximum at 110 °C—where it was 4.51% higher than control and this improvement was statistically significant (*P* < 0.05)—before declining at higher temperatures. These textural changes can be explained by the structural modifications induced by dry-heat treatment and the compositional impact of quinoa flour. The reduction in wet gluten content due to quinoa addition inherently weakens the continuity of the gluten network. Moreover, dry-heat treatment promotes the aggregation of denatured proteins, which further impedes the development of a cohesive, ordered three-dimensional matrix during dough development. This compromised network results in poorer gas-holding capacity, leading to a denser crumb structure and increased hardness, particularly at higher treatment temperatures. The initial improvement in springiness at 110 °C may reflect a moderate level of protein aggregation that enhances elastic recovery without severely disrupting network continuity. However, excessive aggregation at elevated temperatures (>130 °C) leads to a brittle, over-aggregated protein phase, which diminishes both springiness and overall loaf quality (González et al., 2020). Thus, the optimal textural profile observed at 110 °C underscores the importance of controlled thermal modification in balancing protein interactions and maintaining desirable bread structure.

### 3.5 Effect of different dry-heat treated quinoa flour on the in vitro digestibility of bread

The in vitro starch digestibility profiles of breads containing quinoa flour subjected to different dry-heat treatments are presented in Table 3. Compared to the control bread, all thermally treated samples showed a significant reduction in rapidly digestible starch (RDS) content, with the minimum value recorded for bread prepared from flour treated at 150 °C. Concurrently, the resistant starch (RS) fraction increased markedly across all treatment temperatures. The content of slowly digestible starch (SDS) initially increased, peaking in bread containing 110 °C-treated flour, before declining at higher temperatures. Consequently, the combined proportion of SDS increased significantly, reaching maximal increments of 6.99% and 2.25% for SDS and (SDS + RS), respectively, in bread made with 110 °C-treated flour. These shifts in starch digestibility are closely linked to the structural reorganization induced by dry-heat treatment. The reduction in RDS can be primarily ascribed to the physical barrier created by protein aggregates and the enhanced crystallinity of starch granules, both of which limit the accessibility and catalytic efficiency of amylolytic enzymes. The observed rise in RS is consistent with the formation of more ordered, thermally stabilized starch structures—evidenced by the increased relative crystallinity in XRD analysis—that are inherently less susceptible to enzymatic hydrolysis. The initial increase in SDS at moderate temperatures (110 °C) suggests a transitional structural state in which starch is partially but not fully rearranged into enzyme-resistant conformations. At higher temperatures (>130 °C), the slight decline in SDS may indicate progressive molecular reorganization toward either highly ordered RS or, conversely, disorganized amorphous regions with varied digestibility (González et al., 2020; Zheng et al., 2024). Elevating the SDS and RS fractions is a recognized nutritional target for starchy foods, as it moderates postprandial glycemic response and extends energy release (Morales-Huerta et al., 2025; Oh et al., 2018). The present findings thus demonstrate that dry-heat treatment, particularly at 110 °C, can effectively modulate the starch digestibility of quinoa-enriched bread toward a more nutritionally favorable profile, primarily through physical restructuring rather than chemical alteration. Specifically, the reduction in RDS and the increase in RS are attributable to two physical modifications induced by DHT: (i) the enhanced starch crystallinity (as evidenced by XRD, Fig. 2a), which limits the accessibility of amylolytic enzymes to the glucosidic bonds; and (ii) the formation of surface protein barriers (as observed by SEM, Fig. 1), which physically hinders enzyme diffusion and binding to starch granules (González et al., 2020).

**Table 3:** Effects of different dry-heat treatment (DHT)on the content of RDS, SDS and RS of quinoa bread.

Samples	RDS/%	SDS/%	RS/%	SDS + RS/%
Control	50.12 ± 0.19^a^	25.90 ± 0.18^d^	23.98 ± 0.26^d^	49.88 ± 0.42^d^
110 °C	49.00 ± 0.12^ab^	27.71 ± 0.14^a^	23.29 ± 0.18^c^	51.00 ± 0.32^bc^
130 °C	48.04 ± 0.12^bc^	26.34 ± 0.23^bc^	25.62 ± 0.22^b^	51.96 ± 0.40^b^
150°C	47.68 ± 0.21^d^	26.89 ± 0.25^b^	25.43 ± 0.19^a^	52.32 ± 0.78^a^

Control, untreated quinoa flour; 110 °C, 130 °C, and 150°C denote quinoa flour dry-heat treated at 110 °C, 130 °C, and 150 °C for 1 h, respectively. Values are expressed as mean ± SD (*n* = 3). Different lowercase letters within the same column indicate significant differences among treatment groups (*P* < 0.05, one-way ANOVA followed by Duncan's multiple range test). RDS, rapidly digestible starch; SDS, slowly digestible starch; RS, resistant starch.

### 3.6 Effects of dry-heat-treated quinoa flour on the physicochemical properties of bread

The compositional and functional characteristics of quinoa-enriched bread are summarized in Table 4. Compared to the control bread, the quinoa bread exhibited significantly higher contents of moisture, ash, protein, total dietary fiber, and total phenolics. Notably, total dietary fiber and protein increased by 45.6% and 31.4%, respectively, which substantially enhances the nutritional density of the product. The elevated ash content aligns with findings by Evik and Erta (2019) who reported that quinoa fortification increases the mineral content of foods, attributable to the concentration of minerals in the bran layer of whole quinoa grains. In terms of bioactive components, the total phenolic content in quinoa bread reached 9.97 mg GAE/g, significantly surpassing that of the control. This was accompanied by a DPPH radical scavenging activity of 86.5%, consistent with literature values for quinoa-based baked goods (Yesil & Levent, 2022). The high antioxidant capacity can be explained by two synergistic mechanisms. First, quinoa flour is inherently rich in phenolic compounds, and the thermal processing during baking likely promotes the release of bound phenolics, thereby increasing their extractability and activity. Second, the abundant proteins in quinoa undergo partial thermal degradation into peptides during baking, which can exhibit enhanced antioxidant properties compared to the native proteins (Takatsuka et al., 2022). The combined effect of these soluble phenolics and bioactive peptides contributes to the strong free-radical scavenging activity observed. These results underscore that incorporating dry-heat-treated quinoa flour not only improves the macronutrient profile but also enhances the antioxidant potential of bread, supporting its development as a functional food with added health benefits (Yesil & Levent, 2022). Beyond the physical restructuring of starch, the phenolic compounds released from quinoa flour may exert a complementary biochemical effect by inhibiting intestinal α-glucosidases, thereby retarding glucose absorption (Wang Zhuo, et al., 2025). Additionally, the partial thermal degradation of quinoa proteins into bioactive peptides during baking may also contribute to the overall antioxidant capacity, as reflected by the elevated DPPH radical scavenging activity (Table 4) (Gan et al., 2025). These biochemical factors likely work synergistically with the physical starch modifications to collectively modulate the postprandial glycemic response.

**Table 4:** Effects of dry-heat treatment (DHT) on the physicochemical properties of bread.

Samples	Ash/ (g/100 g)	Moisture/ (g/100 g)	Protein/(g/100 g)	Dietary fiber/(g/100 g)	Total phenolic (mg GAE/g DW)	DPPH radical scavenging activity /%
Control bread	3.34 ± 0.06^b^	5.73 ± 0.17^b^	8.85 ± 0.03^b^	2.72 ± 0.02^b^	3.62 ± 0.14^b^	80.32 ± 3.12^b^
Quinoa bread	3.50 ± 0.04^a^	5.94 ± 0.05^a^	11.63 ± 0.18^a^	3.96 ± 0.09^a^	9.97 ± 0.85^a^	86.50 ± 2.36^a^

Control bread, bread made with 100% wheat flour (no quinoa); Quinoa bread, bread made with quinoa flour (dry-heat treated at 110 °C for 1 h). Values are expressed as mean ± SD (*n* = 3). Different lowercase letters within the same column indicate significant differences between the two bread types (*P* < 0.05).

### 3.7 Effects of dry-heat-treated quinoa flour on metabolic parameters in STZ-induced diabetic mice

**Fig. 4:**
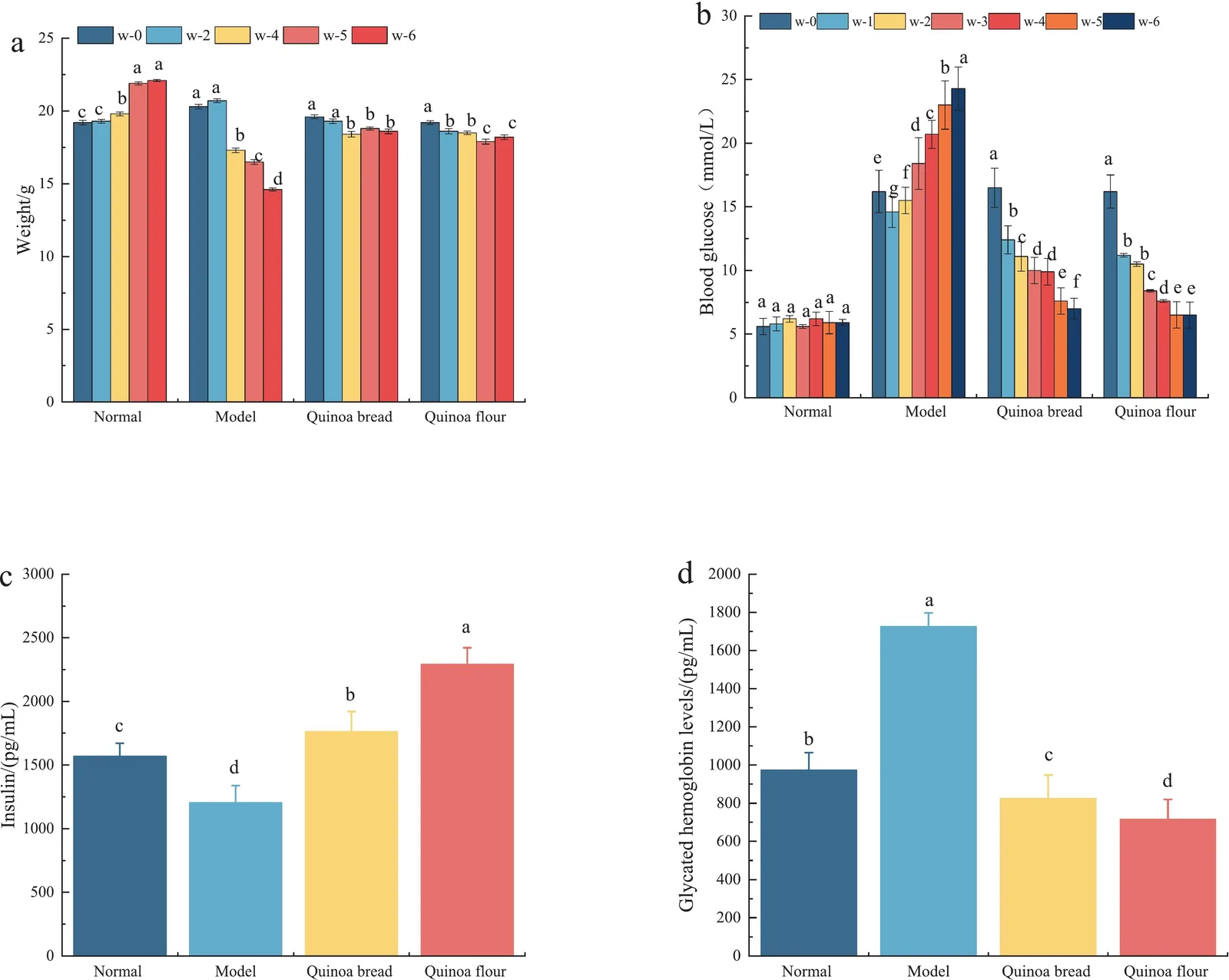
Effects of dietary supplementation with quinoa bread (prepared with 110 °C treated quinoa flour) on metabolic parameters in STZ-induced diabetic mice: (a) body weight, (b) fasting blood glucose, (c) fasting insulin, and (d) glycated hemoglobin. Normal group: healthy mice fed standard diet; Model group: diabetic mice fed standard diet; Quinoa bread: diabetic mice fed bread prepared with 110 °C-treated quinoa flour (15% substitution); Quinoa flour: diabetic mice fed dough prepared with 110 °C-treated whole quinoa flour. Data were collected over a 6-week intervention period. Values are expressed as mean ± SEM (*n* = 8 biologically independent mice per group). Different lowercase letters above the bars indicate significant differences among groups at the same time point (*P* < 0.05, ANOVA followed by Duncan's test).

The metabolic effects of quinoa supplementation in diabetic mice are summarized in Fig. 4. After six weeks of intervention, the body weight of the diabetic model group was 21.5% lower than that of the quinoa bread group (Fig. 4a), indicating that quinoa-enriched bread appeared to attenuate attenuated diabetes-associated weight loss. Body weight in the model group was also significantly lower than in both the normal control and quinoa bread groups. Fasting blood glucose (FBG) levels remained persistently elevated in the model group throughout the study, whereas the quinoa bread group exhibited a progressive decline (Fig. 4b). By week 5, FBG in the quinoa bread group and the whole quinoa flour group had decreased by 66.96% and 71.74%, respectively, compared to the model group, falling below the diagnostic threshold for diabetes. These results demonstrate that quinoa supplementation may contribute to improved glycemic control, with whole quinoa flour showing superior efficacy to quinoa-enriched bread, likely due to its higher concentration of bioactive components (López-Moreno et al., 2023). The hypoglycemic activity of quinoa can be attributed to its rich profile of phytochemicals and nutrients. Quinoa is abundant in phenolic compounds, which may inhibit polysaccharide-digesting enzymes (e.g., α-amylase and α-glucosidase), thereby slowing starch hydrolysis and reducing postprandial glucose influx. Furthermore, its high dietary fiber content can delay gastric emptying, modulate carbohydrate absorption, and improve insulin sensitivity. The elevated protein content may also contribute to prolonged satiety and slower nutrient digestion, collectively supporting better glycemic control (Eman Alamri et al., 2023).

As shown in Fig.4c, fasting insulin (FINS) levels were significantly lower in the model group than in normal controls. Quinoa bread and whole quinoa flour supplementation increased FINS by 46.31% and 90.26%, respectively, suggesting a potential improvement in pancreatic β-cell function or enhanced insulin secretion. Glycated hemoglobin (GHb), a marker of long-term glycemic control, was elevated by 77.29% in the model group relative to normal controls (Fig. 4d). Quinoa bread and whole quinoa flour reduced GHb by 52.18% and 58.49%, respectively, reinforcing the sustained glucose-lowering effect of quinoa. The observed improvements in insulin and GHb are consistent with the modulation of dietary fiber and polyphenols on insulin signaling and glucose metabolism. In summary, dietary supplementation with quinoa, particularly in the form of whole quinoa flour, significantly ameliorates hyperglycemia, improves insulin levels, and mitigates weight loss in STZ-induced diabetic mice. These benefits are likely mediated through multiple mechanisms involving enzyme inhibition, delayed carbohydrate digestion, and enhanced insulin sensitivity, supporting the potential of quinoa as a functional ingredient for diabetes management. Integrating the in vitro and in vivo findings, the observed hypoglycemic effect is likely the result of synergistic interactions between physical and biochemical mechanisms (González et al., 2020; Wang Zhuo, et al., 2025; Zhou et al., 2020). Notably, given that the shift in starch digestibility fractions (RDS↓, RS↑) was more pronounced than the increase in phenolic content, we propose that starch structural modification plays a dominant role in the acute glycemic response, while phenolic compounds may contribute to longer-term antioxidant and insulin-sensitizing effects. Nevertheless, the current experimental design does not allow us to quantitatively deconvolute the relative contributions of these two mechanisms, which represents a limitation of this study. Future investigations using isolated starch or specific enzyme-inhibitor assays are warranted to further distinguish between physical and biochemical contributions to the overall hypoglycemic effect.

It should be acknowledged that the diets administered to the quinoa flour and quinoa bread groups were not isocalorically matched to the standard diet, which may introduce a potential confounding factor. Nevertheless, future studies with isocaloric diet designs would be valuable to further confirm the conclusions drawn from the present work.

## 4 Conclusion

In summary, dry-heat treatment (DHT) of whole quinoa flour, particularly at 110 °C, effectively modified its structure by detaching surface proteins and increasing starch crystallinity, while preserving its chemical integrity. These structural changes led to improved dough water absorption, an optimized bread texture characterized by significantly enhanced springiness at 110 °C (*P* < 0.05) and a slight, though not statistically significant, reduction in hardness, and a nutritionally favorable starch profile characterized by decreased rapidly digestible starch and increased slowly digestible and resistant starch fractions. In the in vivo validation using the 110 °C optimized treatment, bread incorporating DHT-treated quinoa flour suggested potential improvements in metabolic parameters in STZ-induced diabetic mice, including body weight, blood glucose, and insulin levels, thereby confirming the hypoglycemic potential predicted by the in vitro screening. These findings suggest that DHT is a viable physical strategy to enhance the technological functionality of quinoa in baked goods, and that quinoa bread supplementation may contribute to improved glycemic control in this STZ-induced diabetic model. Several limitations of this study should be acknowledged. First, only one quinoa substitution level (15%) and one DHT duration (1 h) were evaluated, and the optimal temperature (110 °C) was selected based on in vitro criteria. Second, no sensory evaluation or human glycemic index testing was conducted, which would be necessary to translate these findings to practical food applications. Third, the in vivo mechanistic biomarkers were limited to FBG, insulin, and GHb, without assessing glucose tolerance, insulin resistance indices, pancreatic histology, or gut microbiota composition. Further studies incorporating glucose tolerance tests, insulin resistance indices, and pancreatic histology are warranted to fully elucidate the underlying mechanisms.

The following are the supplementary data related to this article.

Supplementary Table S1

## CRediT authorship contribution statement

**Ya-li Zhou:** Writing – original draft, Visualization, Validation, Supervision, Methodology, Investigation, Funding acquisition, Formal analysis, Data curation, Conceptualization. **Xiao-long Li:** Writing – original draft, Visualization, Validation, Methodology, Investigation, Formal analysis, Data curation. **Xin-yong You:** Writing – original draft, Methodology, Investigation, Formal analysis, Data curation, Conceptualization. **Xing-yun Wang:** Writing – review & editing.

## Informed consent statement

Not applicable

## Ethics statement

All animal procedures were conducted in accordance with the Guidelines for the Care and Use of Laboratory Animals. Male C57BL/6 mice were supplied by Henan Kebes Biotechnology Co., Ltd. (Laboratory Animal Production License No. SCXK (Yu) 2020–0005). The experimental protocol was reviewed and approved by the Institutional Animal Care and Use Committee of the Institute of Radiation Medicine, Chinese Academy of Medical Science (Approval No. IRM-DWLL-2022250). All efforts were made to minimize animal suffering and to reduce the number of animals used, in compliance with the 3Rs principle (Replacement, Reduction and Refinement).

## Funding

This study was supported by the Henan Provincial Scientific and Technological Project (252102110074), the Major Science and Technology Special Project of Anyang, China (2025A02NY001), the Natural Science Foundation of Henan (242300421553), and the Scientific Research Start-up Funds of Anyang Institute of Technology (BSJ2020019).

## Declaration of competing interest

The authors declare that they have no known competing financial interests or personal relationships that could have appeared to influence the work reported in this paper.

## Data Availability

Data will be made available on request.
