# Supplementary material for: From raw material to functional food: Effect of dry-heat treatment on whole quinoa flour structure and dough rheology, with in vivo hypoglycemic validation at the optimal temperature
Source: Food Chem X. 2026 Aug 30;39:104360. doi: 10.1016/j.fochx.2026.104360 (PMC13563555; doi:10.1016/j.fochx.2026.104360)
Supplement: Supplementary file 1 [file mmc1.docx]

Table S1 Relative crystallinity of different dry-heat treatment quinoa flour samples

|  | Bck | B110 | B130 | B150 |
| --- | --- | --- | --- | --- |
| the relative crystallinity /% | 38.56±0.38^d^ | 41.35±0.38^c^ | 42.68±0.38^ab^ | 43.21±0.38^a^ |

Bck: untreated quinoa flour; B110: quinoa flour dry‑heat treated at 110°C for 1 h; B130: quinoa flour dry‑heat treated at 130°C for 1 h; B150: quinoa flour dry‑heat treated at 150°C for 1 h. Values are expressed as mean ± standard deviation (n=3); different letters indicate significant differences (P < 0.05).
